# The Effects of the Honey-Roasting Process on the Pharmacokinetics of the Six Active Compounds of Licorice

**DOI:** 10.1155/2018/5731276

**Published:** 2018-06-21

**Authors:** Yulong Zhang, Mengyue Wang, Jingyu Yang, Xiaobo Li

**Affiliations:** ^1^School of Pharmacy, Shanghai Jiao Tong University, Shanghai, China; ^2^College of Traditional Chinese Materia Medica, Shenyang Pharmaceutical University, Shenyang, China

## Abstract

A convenient UPLC-MS/MS method was established to determine the contents of six bioactive compounds, namely, liquiritin apioside, liquiritin, isoliquiritin, liquiritigenin, isoliquiritigenin, and glycyrrhetinic acid, in rat plasma and their pharmacokinetics. By comparing the pharmacokinetic parameters of these compounds in rats by orally administering raw and honey-roasting licorice, the C_max_ of isoliquiritin showed a significant decrease, while the AUC_0-24h_ showed no significant differences. The C_max_ and AUC_0-24h_ of isoliquiritigenin were increased by 49.3% and 42.7% over those of the raw licorice group, respectively. These results indicate that the absorption of isoliquiritin in rats was reduced while the absorption of isoliquiritigenin was promoted in the honey-roasting process. These results may provide one explanation as to why licorice is more able to relieve cough, while honey-roasting licorice is better at invigorating* qi *and restoring pulse. Furthermore, the C_max_ of glycyrrhetinic acid was increased, suggesting that it may enhance the tonic effect of licorice. Additionally, the amount of honey added in the honey-roasting process influenced the pharmacokinetic parameters of the six compounds whose absorption decreased when the 50% honey-roasting licorice water decoction was administered. These results provide an experimental basis for studying the influence of licorice processing on bioactive compound pharmacokinetics.

## 1. Introduction

Licorice, Gancao in Chinese, is a commonly used traditional Chinese medicine (TCM) and is frequently divided into raw Gancao (Glycyrrhizae Radix et Rhizoma) and Zhi-gancao (Glycyrrhizae Radix et Rhizoma Praeparata Cum Melle) in TCM formularies. Zhi-gancao is produced by a method in which raw Gancao is stir-fried with honey until it becomes yellow to deep yellow and not sticky and is then cooled in the air [1]. The uses of raw licorice could reinforce the function of the* spleen*, dispel* phlegm*, relieve cough, and moderate the properties of other herbs; however, Zhi-gancao is used to invigorate the functions of* spleen* and stomach as well as to reinforce* qi* and promote blood circulation [1]. It was reported that honey-roasting licorice (Zhi-gancao) also has better anti-inflammatory [2, 3], neuroprotective [4], and immunity-enhancing [5] properties than raw licorice.

Chemical and pharmaceutical studies showed that triterpene saponins and flavonoids [6, 7] are the main bioactive compounds in licorice. Liquiritin can stimulate immune responses and activate antioxidant enzymes [8]; liquiritigenin and isoliquiritigenin display PPAP*γ* activating activity, suggesting their potential as a treatment of metabolic syndrome [9]. Moreover, glycyrrhetinic acid, a metabolic product of glycyrrhizic acid [10], was reported to have positive effects on the protection of rat hepatocytes [11] and the inhibition of inflammation [12].

A great deal of research has focused on the pharmacological effects of raw and honey-roasting licorice. The different processing technologies are associated with variations in the chemical constituents of licorice [13]. We have shown that after honey-roasting, the contents of the effective chemical components varied, with lessening of the decocting quantity of liquiritin apioside and liquiritin and increase in isoliquiritin [14–16]. The pharmacokinetic parameters of bioactive flavones were detected in rat plasma after oral administration of flavone-enriched raw licorice [17]. Few studies on the pharmacokinetic profiles of the honey-roasting licorice have been performed. Hence, after oral administration of honey-roasting licorice, to detect bioactive compounds in rat plasma and their pharmacokinetic behaviors is worth studying to explain the medicinal principles which are responsible for the pharmacological effects of honey-roasting licorice.

Therefore, this study aims to compare the pharmacokinetics of liquiritin apioside, liquiritin, isoliquiritin, liquiritigenin, isoliquiritigenin, and glycyrrhetic acid in rats after oral administration of raw/honey-roasting licorice. The results of this study provide helpful information to facilitate the clinical application and processing technology of honey-roasting licorice.

## 2. Materials and Methods

### 2.1. Chemicals and Reagents

HPLC grade acetonitrile was purchased from Merck Company (Darmstadt, Germany). Ultrapure water for the UPLC mobile phase was prepared using a Milli-Q purification system (Millipore, Bedford, MA, USA). Raw licorice slices were purchased from Yili Science and Technology Industry Co., Ltd. (Inner Mongolia, China). Honey was purchased from Jing'an Pharmaceutical Co., Ltd. (Shanghai, China). Honey-roasting licorice slices were prepared in the lab (the details of the experiment are given in* Licorice Processing and Decoction*). The reference standards of liquiritin apioside (LA), liquiritin (LQ), isoliquiritin (ILQ), liquiritigenin (LG), isoliquiritigenin (ILG), glycyrrhetinic acid (GA), and andrographolide (internal standard, IS) were purchased from Sichuan Wekeqi Co., Ltd. (Sichuan, China). Other chemicals were of analytical grade.

### 2.2. Licorice Processing and Decoction

In accordance with the Chinese Pharmacopoeia Committee (2015), raw licorice slices (100 g for each) were stir-fried with 25 g of honey until they became yellow to deep yellow and were not sticky to the fingers. They were cooled in the air to yield 25% honey-roasting licorice (w/w) slices. With the same method, 50% honey-roasting licorice (w/w) slices were prepared.

Raw licorice slices (100 g) were decocted twice in distilled water (1000 mL) for 30 min. Water decoction of raw licorice was filtered and concentrated to 1.00 g (crude drug)/mL using a rotary evaporation apparatus. The 25% honey-roasting licorice slices (100 g, equivalent to 87 g raw licorice) and 50% honey-roasting licorice slices (100 g, equivalent to 72 g raw licorice) were treated with the same method.

### 2.3. Animals

Healthy male Wistar rats (weighing 200 ± 20 g) were obtained from the Laboratory Animal Centre of Shanghai Jiao Tong University (Shanghai, China). The rats were kept in a temperature-controlled environment with free access to laboratory food and water for one week and were deprived of food for 12 h before the experiment. All protocols and procedures were approved by the Shanghai Jiao Tong University Animal Care and Use Committee.

### 2.4. Chromatographic and Mass Spectrometric Conditions

Quantification of LA, LQ, ILQ, LG, ILG, and GA was performed on a Waters UPLC system with an Applied Biosystem 5500 QTRAP® hybrid triple-quadrupole mass spectrometer (Applied Biosystems/MDS Sciex, Foster City, CA, USA), equipped with a turbo ion spray source. Chromatographic separation was performed on a ZORBAX Eclipse Plus C18 column (50 mm × 2.1 mm, 1.8 *µ*m). The mobile phase consisted of 2 mM ammonium acetate water (A) and acetonitrile (B) using a gradient elution of 10%-95% B at 0-4.0 min, 95% B at 4.0-5.0 min, 95%-10% B at 5.0-5.1 min, and 10% B at 5.1-7.0 min. The flow rate was set at 0.4 mL/min. The column temperature and injection volume were set at 40°C and 2.0 *µ*L, respectively. An MS system operating in negative electrospray ionization mode was employed in this study. Quantification was performed using a multiple reaction monitoring (MRM) model of the transition* m/z* [M-H] ^−^ 549.2→134.9 for LA,* m/z* [M-H] ^−^ 417.2→118.9 for LQ,* m/z* [M-H] ^−^ 417.1→134.9 for ILQ,* m/z* [M-H] ^−^ 255.1→118.9 for LG,* m/z* [M-H] ^−^ 255.2→118.9 for ILG,* m/z* [M-H] ^−^ 469.3→355.3 for GA, and* m/z* [M-H] ^−^ 349.2→287.1 for IS. The DP for each compound and IS were -120, -100, -100, -100, -100, -80, and -100 V, respectively. The collision energy for each compound and IS were -58, -32, -42, -32, -32, -65, and -20 V, respectively. The ion spray needle voltage was set at -4500 V, and the source temperature was 500°C. Following optimization of the setting parameters, the ESI source was operated with the GS1, GS2, and CUR (Nitrogen) set at 35, 35, and 40 psi, respectively.

### 2.5. Stock Solutions and Quality Control Samples

Standard stock solutions of LA, LQ, ILQ, LG, ILG, GA, and IS (1.00 mg/mL, respectively) were dissolved in acetonitrile. The working solutions for the calibration curve were diluted with 70% acetonitrile. The plasma samples for the calibration curve were prepared by spiking the above working solutions into blank plasma to yield the final concentration series: 4.75-800.00 ng/mL for LA, 0.83-500.00 ng/mL for LQ, 0.39-833.33 ng/mL for ILQ, 0.42-20.00 ng/mL for LG, 0.17-500.00 ng/mL for ILG, and 2.37 -500.00 ng/mL for GA.

The IS working solution was also diluted to a concentration of 10.00 *µ*g/mL with 70% acetonitrile. The QC samples at three concentration levels—10.00, 100.00, and 500.00 ng/mL for LA; 1.00, 166.67, and 333.33 ng/mL for LQ; 1.67, 40.00, and 500.00 ng/mL for ILQ; 0.84, 10.00, and 16.67 ng/mL for LG, 0.33, 166.67; and 333.33 ng/mL for ILG, 5.00, 10.00, and 100.00 ng/mL for GA (low, medium, and high levels, respectively)—were prepared in blank plasma.

### 2.6. Sample Preparation

Fifty microliters of the plasma samples (or standard plasma samples and QC samples) were spiked with 5 *µ*L of IS solution (10.00 *µ*g/mL) and 200 *µ*L of acetonitrile. The mixture was vortexed for 30 s and centrifuged at 14,000 rpm for 15 min at 4°C. The supernatant was collected and evaporated in a 37°C water bath under a nitrogen stream. The residue was redissolved with 500 *µ*L of 70% acetonitrile.

### 2.7. Method Validation

The method was validated for selectivity, linearity, lower limit of quantification, accuracy, precision, extraction recovery, and stability according to the US Food and Drug Administration Bioanalytical Method Validation Guide.

#### 2.7.1. Selectivity

The selectivity of the method was investigated by comparing the chromatograms of the blank plasma sample; blank plasma sample spiked with LA, LQ, ILQ, LG, ILG, GA, and IS; and plasma sample after administration of the licorice decoction.

#### 2.7.2. Linearity and Lower Limit of Quantification (LLOQ)

The linear regression equation consisted of five concentration levels and was determined by plotting the ratio of the peak as of the compound to IS (*y*) versus the concentration (*x*) of the reference standard with a 1/x-weighted least-square linear regression algorithm. The LLOQ was defined as the lowest concentration on the calibration curve (signal/noise = 10), determined with accuracy (expressed as relative error, RE) within ± 15% and precision (expressed as relative standard deviation, RSD) less than 15%.

#### 2.7.3. Accuracy and Precision

The intraday accuracy and precision of the method were assessed by six replicate analyses of QC samples at low, medium, or high concentrations on the same day. Similarly, the interday accuracy and precision were determined for three consecutive days. The intra- and interday accuracy were expressed as RE, and the precision was expressed as RSD.

#### 2.7.4. Extraction Recovery and Matrix Effect

The extraction recovery of each analyte was calculated as extraction recovery (%) = (peak area of analyte spiked in blank sample × 100/peak area of analyte spiked in postpreparative sample). The matrix effect was measured by comparing the peak areas of the postextracted standard plasma samples with 70% acetonitrile containing equivalent amounts of all the analytes.

#### 2.7.5. Stability

The stabilities of the six compounds in rat plasma were evaluated by analyzing QC samples under different conditions. Short-term stability was tested by storing the postpreparative QC samples in the autosampler at 4°C for 24 h. Long-term stability was tested by storing QC samples at -80°C for one month. RSD was evaluated by analyzing six replicate samples.

### 2.8. Pharmacokinetic Study

After being housed in a controlled environment for a week, rats were randomly divided into three groups (n = 5 for each group) and were then orally administered a raw, 25% honey-roasting, or 50% honey-roasting licorice water decoction at a dosage of 2.7 g (crude drug)/kg. Blood samples of 0.15 mL were taken from the suborbital vein into heparinized tubes at 0.17, 0.5, 1, 1.5, 2, 2.5, 3, 4, 6, 8, 12, and 24 h after oral administration of the licorice water decoction. The blood samples were immediately centrifuged at 10,000 rpm for 15 min. The separated plasma samples were frozen at -80°C before analysis.

### 2.9. Statistical Analysis

The pharmacokinetic parameters of the six compounds were calculated by KINETICA 4.4.1 (Thermo Electron Corporation, Philadelphia, PA, USA) software. Noncompartmental analysis was used to determine the area under the curve (AUC_0-24h_), maximum plasma concentration (C_max_), and time to attain (T_max_). Data were presented as the mean ± SD. Statistical analyses of all the data obtained were evaluated using one-way ANOVA (SPSS 17.0).

## 3. Results and Discussion

### 3.1. Method Validation

#### 3.1.1. Selectivity

**Figure 1:**
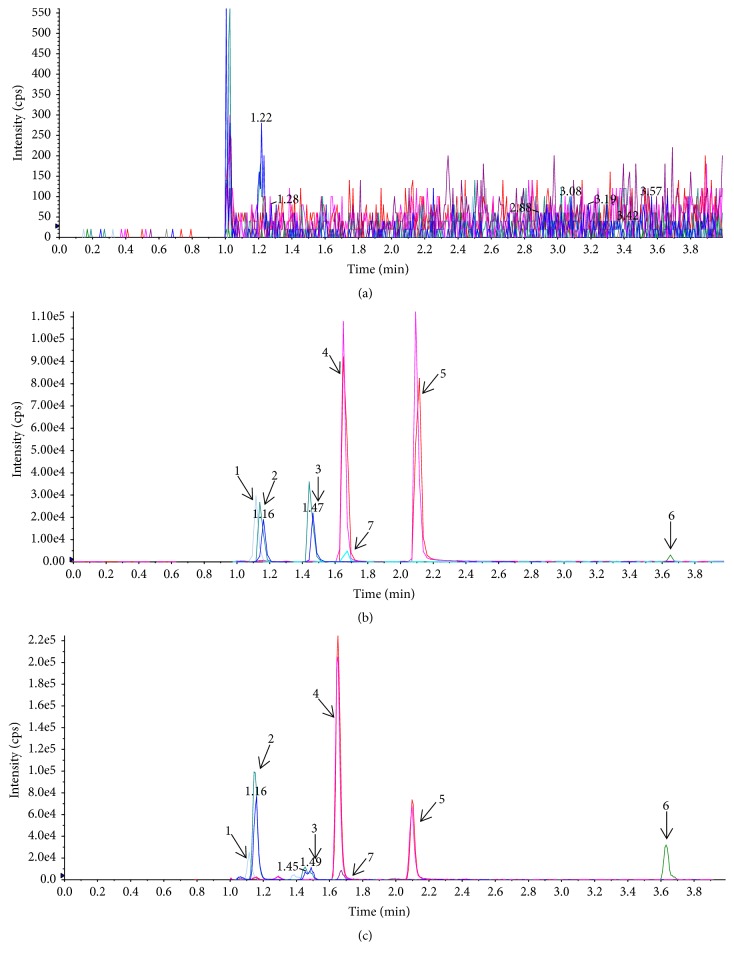
Representative MRM chromatograms of LA, LQ, ILQ, LG, GA, and IS in plasma: (a) blank plasma; (b) a blank plasma sample spiked with LA (100.00 ng/mL), LQ (166.67 ng/mL), ILQ (40.00 ng/mL), LG (16.67 ng/mL), ILG (166.67 ng/mL), GA (5.00 ng/mL) reference standard, and IS (10.00 *µ*g/mL); (c) and an actual plasma sample obtained from a rat at 2.5 h after oral administration of 25% honey-roasting licorice. 1 (LA), 2 (LQ), 3 (ILQ), 4 (LG), 5 (ILG), 6 (GA), 7 (IS).

LA, LQ, ILQ, LG, ILG, and GA were identified in rat plasma by UPLC-MS after oral administration of raw/honey-roasting licorice. The selectivity of the method was determined by comparing representative chromatograms of blank rat plasma, blank rat plasma spiked with six reference standards (LA, LQ, ILQ, LG, ILG, and GA) and andrographolide, and plasma samples after oral administration of 25% honey-roasting licorice (Figure 1). It was shown that no interference was detected from endogenous substances and the background noise was low.

#### 3.1.2. Linearity and Lower Limit of Quantification (LLOQ)

The linear regression equations, linear ranges, and LLOQs of the six compounds are listed in Table 1. The linear regression equations of all of the compounds exhibited good linearity with correlation coefficients (r^2^) > 0.99. The LLOQs for LA, LQ, ILQ, LG, ILG, and GA were appropriate for the quantitative detection of these compounds in pharmacokinetic studies.

#### 3.1.3. Precision and Accuracy

The precision and accuracy were determined to analyze the quality control (QC) samples at three concentration levels. The intraday precision (n = 6, RSD%) and accuracy (n = 6, RE%) of the six compounds ranged from 2.8% to 10.5% and 85.1% to 103.6%, respectively. The interday precision (n = 6, RSD%) and accuracy (n = 6, RE%) ranged from 3.9% to 12.4% and 86.2% to 102.3%, respectively. The precision and accuracy data are summarized in Table 2. These results indicated that the method was reproducible and accurate for detecting the six compounds in rat plasma.

#### 3.1.4. Extraction Recovery and Matrix Effect

The extraction recoveries of the six compounds at three QC concentration levels are shown in Table 2. These data indicated that the extraction efficiency of the method was within an acceptable range. The matrix effect values obtained for all analytes ranged from 101.1% to 109.1%, and the matrix effect for IS was 104.3%. The results suggested that the matrix effects for all analytes and IS were in acceptable range.

#### 3.1.5. Stability

The stability of the six compounds was evaluated under various conditions. QC samples stored in a freezer at -80°C remained stable for one month. QC samples after preparation in an autosampler (4°C) for 24 h appeared to have no effect on the quantitation of the six compounds. The stability data were within the acceptance range of 85%-115% (Table 3). The results showed that, in rat plasma, the six compounds were stable before quantitation.

### 3.2. Comparative Pharmacokinetic Studies

**Figure 2:**
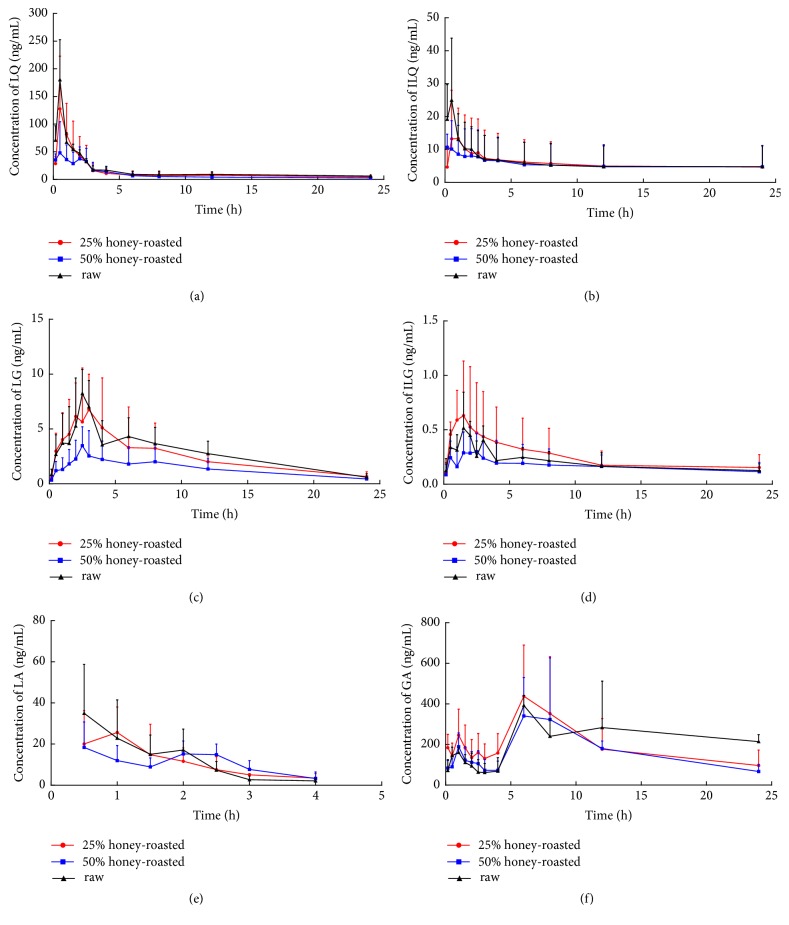
Plasma concentration-time profiles of LQ (a), ILQ (b), LG (c), ILG (d), LA (e), and GA (f) after oral administration water decoction of 25% honey-roasting licorice, 50% honey-roasting licorice, and raw licorice, respectively.

The validated UPLC-MS/MS method was applied to the pharmacokinetic study of LA, LQ, ILQ, LG, ILG, and GA in rats after oral administration of a water decoction of raw or honey-roasting licorice. The concentration-time curves of the six compounds in rat plasma are shown in Figure 2, and the corresponding pharmacokinetic parameters are summarized in Table 4. Comparison of the pharmacokinetic parameters of five flavones in rats after oral administration of raw/honey-roasting licorice showed that the C_max_ and AUC_0-24h_ of LA, LQ, ILQ, and LG decreased in honey-roasting licorice. Additionally, the C_max_ and AUC_0-24h_ of GA were higher than those in raw licorice group, but there were no significant differences.

First, the C_max_ of flavones was reduced in the honey-roasting groups; the C_max_ of ILQ in the 25% honey-roasting group was 24.80 ± 3.63 ng/mL, which was 48.07% lower than that in the raw group. Additionally, T_max_ was 1.75 times longer in 25% honey-roasting group. However, there were no significant differences between the 25% honey-roasting and raw groups regarding AUC_0-24h_. These results showed that ILQ was absorbed by rats more slowly after the honey-roasting treatment. Meanwhile, after processing with honey, the decocted contents of the main glycosides of licorice obviously changed. Our previous studies showed that the quantity of ILQ from water decoction of honey-roasting licorice was higher than that of licorice [14–16]. Thus, the honey-roasting process affected the pharmacokinetic behavior of ILQ, which can be antispasmodic [18] and antitussive [19]. These results provide supportive evidence that explains why raw licorice is more able to alleviate pain and relieve cough than honey-roasting licorice. While the AUC_0-24h_ of ILG was significantly improved (P<0.05) from 6.53 ± 2.34 h·ng/mL to 9.32 ± 0.68 h·ng/mL after licorice was processed with honey, the C_max_ in the 25% honey-roasting group was 49.3% higher than that in raw group for ILG. Additionally, ILG was reported to have an antiplatelet effect [20] and an accumulation of cAMP [21]. This suggested that ILG could alleviate symptoms, such as insufficiency of the* spleen* and deficiency of* qi* and blood, by increasing the accumulation of cAMP in the rat ventricular heart muscle, thus improving circulation. We guess that ILG was probably the bioactive compound, which was thought to be related to the honey-roasting licorice invigoration of* qi* and promotion of blood circulation.

Another bioactive compound of licorice, glycyrrhizin, was reported to have extremely low oral bioavailability [22, 23]. Glycyrrhizin was mainly absorbed as glycyrrhetinic acid after metabolism by intestinal bacteria [24]. Thus, only glycyrrhetinic acid was detected in rat plasma in this study. The C_max_ and AUC_0-24h_ of GA in the 25% honey-roasting group were higher than those in the raw group. Interestingly, the C_max_ and AUC_0-24h_ of GA were remarkably higher than the total of the five flavones. Additionally, GA has been reported to have a hepatoprotective effect [25] and modulate inflammatory markers [26]. Based on these results, we suggest that GA in licorice might be the key to the efficacy of honey-roasting for invigorating* qi* and restoring pulse.

Furthermore, the C_max_ and AUC_0-24h_ of LQ, LG, and ILG between the 25% honey-roasting and 50% honey-roasting groups exhibited a great change. Moreover, compared with the raw licorice group, the C_max_ of LA, LQ, and LG were 43.7%, 53.96%, and 54.9% lower in the 50% honey-roasting group, respectively. There were no significant differences in the pharmacokinetic parameters for LA, LQ, LG, and ILG between the raw and 25% honey-roasting groups. The most possible cause of these phenomena is that the quantity of honey affected the contents of bioactive compounds in licorice. Previous research has shown that the contents of LQ and LG in honey-roasting licorice decreased with the amount of honey added during the honey-roasting process [27]. In addition, the content of LQ is in accordance with the quality standard of licorice listed in the Chinese Pharmacopoeia Committee (2015). In conclusion, after licorice was processed with honey, the contents of flavones varied, which influenced the pharmacokinetic behavior of the flavones in rats fed honey-roasting licorice. Therefore, the amount of honey added during the honey-roasting process should be strictly controlled.

## 4. Conclusions

In this study, the comparison of the pharmacokinetic behaviors of licorice flavones indicated that the absorption of isoliquiritin was inhibited while that of isoliquiritigenin was promoted after the honey-roasting process. Additionally, the amount of honey added during the honey-roasting process influenced the pharmacokinetic parameters of the six compounds. These findings prove that honey-roasting process can influence the pharmacokinetic behaviors of bioactive compound in licorice and give one possible explanation of the phenomena that raw licorice is stronger at relieving cough, while honey-roasted licorice is better at invigorating* qi* and restoring pulse.

## Figures and Tables

**Table 1:** Linear regression equation, linear range, and LLOQ of six compounds.

Compound	Linear Regression Equation	r^2^	Linear range (ng/mL)	LLOQ (ng/mL)
LA	y =0.1056x + 0.2601	0.9981	4.75 - 800.00	4.75
LQ	y =0.0447x - 0.1266	0.9973	0.83 - 500.00	0.83
ILQ	y = 6.6859x + 1.256	0.9988	0.39 - 833.33	0.39
LG	y = 0.9577x - 0.2405	0.9952	0.42 - 20.00	0.42
ILG	y = 5.3311x - 0.6098	0.9966	0.17 - 500.00	0.17
GA	y = 6.0739x – 1.9631	0.9997	2.37 - 500.00	2.37

**Table 2:** Precision, accuracy, and extraction recovery of six compounds (n = 6).

Compound	Intra-day		Inter-day		Recovery		Matrix Effect	
Concentration (ng/mL)	Precision RSD (%)	Accuracy RE (%)	Precision RSD (%)	Accuracy RE (%)	Average (%)	RSD (%)	Average (%)	RSD (%)
LA								
500	10.5	86.7	12.4	89.7	76.9	5.2	102.1	9.6
40	8.3	91.3	9.8	92.1	78.7	6.7	106.7	7.6
10	6.8	88.2	5.7	87.9	82.1	4.3	102.9	7.5
LQ								
333.33	5.2	95.0	9.7	102.3	96.3	9.5	106.3	7.5
16.67	9.1	97.9	4.6	94.7	90.2	10.3	101.6	5.3
0.83	4.6	85.1	3.9	96.9	89.6	8.6	101.1	6.3
ILQ								
833.33	5.2	100.8	9.0	91.4	79.8	4.6	108.4	2.0
166.67	7.4	95.2	7.3	94.2	85.2	5.7	106.6	12.2
1.67	8.7	89.1	6.5	90.3	94.3	7.4	104.3	4.7
LG								
166.67	4.6	101.5	6.8	95.4	97.6	5.9	103.9	10.1
8.34	3.9	98.6	4.9	94.9	90.4	4.5	106.6	7.9
0.42	2.8	102.4	5.2	94.7	93.8	7.6	109.1	6.7
ILG								
333.33	5.1	87.4	8.6	87.7	77.7	6.4	108.4	11.6
16.67	7.6	92.5	7.3	86.2	87.4	5.8	105.6	7.5
0.33	4.2	95.8	4.4	93.8	80.1	5.4	104.4	9.6
GA								
1000	7.6	103.6	8.7	98.7	97.1	7.3	107.7	5.3
100	5.4	95.1	6.5	100.4	85.5	4.6	104.3	13.1
10	3.9	93.4	4.7	94.2	90.3	5.3	104.1	4.2

**Table 3:** Stability of six compounds in rat plasma (n = 6).

Compound	Concentration (ng/mL)	At 4°C for 24 h		At -80°C for 1 month	
		Average (%)	RSD (%)	Average (%)	RSD (%)
LA	500	98.3	4.9	95.3	8.7
	40	92.4	8.6	93.7	9.4
	10	97.3	4.8	93.2	3.8
LQ	333.33	99.5	7.5	89.5	4.9
	16.67	88.3	5.6	95.6	5.3
	0.83	90.9	6.3	96.4	9.5
ILQ	833.33	95.7	8.9	100.1	9.8
	166.67	89.3	3.6	94.2	5.6
	1.67	90.2	7.3	89.4	5.8
LG	166.67	93.9	8.1	96.5	2.1
	8.34	87.7	5.9	97.3	7.8
	0.42	90.8	6.7	92.8	6.5
ILG	333.33	93.1	7.8	87.6	8.2
	16.67	96.4	8.9	85.3	9.1
	0.33	95.7	12.3	94.7	7.5
GA	1000	100.9	11.2	97.3	4.9
	100	96.2	6.7	102.1	7.4
	10	96.4	8.9	96.2	5.0

**Table 4:** Pharmacokinetic parameters for six compounds in rat plasma after oral administration of raw/honey-roasting licorice (n = 5).

Analytes	Group	C_max_ (ng/mL)	T_max_ (h)	AUC_0-24h_ (h·ng /mL)
LA	25% honey-roasting	38.49 ± 13.09	0.8	64.77 ± 17.97^*∗*^
	50% honey-roasting	31.21 ± 8.09^*∗*^	0.9	56.30 ± 14.34
	Raw	55.46 ± 13.51	0.7	82.31 ± 19.00
LQ	25% honey-roasting	202.93 ± 54.12	0.6	478.69 ± 65.22
	50% honey-roasting	87.93 ± 8.08^*∗*Δ^	0.5	318.94 ± 49.99^*∗*Δ^
	Raw	191.00 ± 80.23	0.6	520.48 ± 94.53
ILQ	25% honey-roasting	24.80 ± 3.63^*∗*^	0.7	260.44 ± 8.53
	50% honey-roasting	20.59 ± 5.82^*∗*^	0.4	246.22 ± 7.67^Δ^
	Raw	47.76 ± 2.62	0.4	257.39 ± 19.28
LG	25% honey-roasting	12.02 ± 3.67	2.3	85.36 ± 14.70
	50% honey-roasting	5.46 ± 1.61^*∗*Δ^	2.5	47.74 ± 8.03^*∗*Δ^
	Raw	12.11 ± 5.07	2.4	84.29 ± 33.71
ILG	25% honey-roasting	1.12 ± 0.25	1.5	9.32 ± 0.68^*∗*^
	50% honey-roasting	0.56 ± 0.12^Δ^	1.8	6.13 ± 0.80^Δ^
	Raw	0.75 ± 0.28	1.5	6.53 ± 2.34
GA	25% honey-roasting	650.93 ± 199.95	6.8	6995.30 ± 1293.79
	50% honey-roasting	607.78 ± 277.70	6.8	5177.10 ± 2540.93
	Raw	571.98 ± 311.21	8.0	6979.29 ± 4803.95

*∗P*<0.05, compared with the raw group. Δ*P*<0.05, compared with the 25% honey-roasting group. n: the number of rats.

## Data Availability

This article provides the results of the statistical data, and the primary data could be requested from the corresponding author.
